# Molecular epidemiology of nontuberculous mycobacteria isolated from tuberculosis-suspected patients

**DOI:** 10.1186/s13568-023-01557-4

**Published:** 2023-05-18

**Authors:** Samira Tarashi, Fatemeh Sakhaee, Morteza Masoumi, Morteza Ghazanfari Jajin, Seyed Davar Siadat, Abolfazl Fateh

**Affiliations:** 1 Department of Mycobacteriology and Pulmonary Research, Pasteur Institute of Iran, Tehran, Iran; 2 Microbiology Research Center (MRC), Pasteur Institute of Iran, Tehran, Iran

**Keywords:** Nontuberculous mycobacteria, Clinical significance, *Mycobacterium simiae*, *Mycobacterium jacuzzii*, *Mycobacterium canariasense*, *Mycobacterium chelonae*

## Abstract

It is a growing problem around the world to deal with nontuberculous mycobacteria infection (NTM), but its clinical significance is still largely unknown. This study aims to investigate the epidemiology of NTM infections from various clinical samples and determine their clinical significance. From December 2020 to December 2021, 6125 clinical samples were collected. In addition to phenotypic detection, genotypic detection through multilocus sequence typing (*hsp65*, *rpoB*, and *16S rDNA* genes) and sequencing was also conducted. Records of patients were consulted for clinical information, such as symptoms and radiological findings. Of the 6,125 patients, 351 (5.7%) were positive for acid-fast bacteria (AFB). Out of 351 AFB, 289 (82.3%) and 62 (17.7%) subjects were identified as *M. tuberculosis* complex (MTC) and NTM strains, respectively. Isolates of *Mycobacterium simiae* and *M. fortuitum* were the most frequent, followed by isolates of *M. kansasii* and *M. marinum*. We also isolated *M. chelonae*, *M. canariasense*, and *M. jacuzzii*, which are rarely reported. Symptoms (*P* = 0.048), radiographic findings (*P* = 0.013), and gender (*P* = 0.039) were associated with NTM isolates. *M. Fortuitum*, *M. simiae*, and *M. kansasii* presented with bronchiectasis, infiltration, and cavitary lesions most frequently, while cough was the most common symptom. In conclusion, *Mycobacterium simiae* and *M. fortuitum* were presented in seventeen and twelve NTM isolates from the collected samples. There is evidence that NTM infections in endemic settings may contribute to the dissemination of various diseases and the control of tuberculosis. In spite of this, further research is needed to evaluate the clinical significance of NTM isolates.

## Introduction

Increasing numbers of nontuberculous mycobacteria (NTM) have been isolated from environmental and clinical samples worldwide (Mbeha et al. 2019). NTM agents are ubiquitous environmental saprophytes that are frequently isolated from water, plants, and soil samples (Jeon 2019). Over 200 NTM species have been identified, and new species are being discovered every year (Falkinham 2021). Depending on their growth rate, NTM can be classified as ‘‘slow’’ or ‘‘rapid.’’ Different NTM species are split based on the growth rate, rapid (*e.g*., *M. abscessus*, *M. chelonae*, *M. fortuitum*), and slow growers (*e.g*., *M. avium complex*, *M. kansasii*). Besides, *M. marinum* was introduced as an intermediate category between rapid and slow growers (Huang et al. 2020; Mortazavi et al. 2019). As opportunistic pathogens, NTM agents often cause serious infections such as pulmonary disorders, soft tissue infections, and skin infections. Therefore, it can be regarded as one of the most alarming sources of infection in the healthcare system for both immune-compromised and immune-competent people (Abubakar et al. 2018). Despite surviving in diverse environmental conditions, these microaerobic organisms are extremely antibiotic and disinfectant resistant. Based on these characteristics, NTMs cause a wide variety of infections throughout the world, and hence their epidemiological surveillance is crucial (Kaelin et al. 2020). It is possible to ignore such infections and treat them ineffectively because of misdiagnoses (Ratnatunga et al. 2020). Because NTM infections have similar symptoms to tuberculosis, diagnosis can be challenging (Gharbi et al. 2019; Hirabayashi et al. 2019). It has been reported in several studies from developed countries that the prevalence of NTM infections is increasing (Chunfang et al. 2021; Harada et al. 2021; Winthrop et al. 2020). As a result of the development of molecular methods for detecting NTM infections causality, such as *16SrDNA* sequencing, the reporting rate of NTM infections has gone up in such areas. A lack of laboratory facilities and misdiagnosis may be the cause of this issue in many economically challenged countries (Nasiri et al. 2015; Shafipour et al. 2021). There is still a challenge to detect NTM infections among tuberculosis (TB) suspected samples and to control them in some of these developing countries like Iran. Meanwhile, conducting comprehensive epidemiological studies have been still required across Iran's various regions. To more clearly identify the causative NTM species in this region, epidemiological data were collected from Iranian patients suffering from various NTM infections.

## Materials and methods

### Sample collection and preparation

We conducted a cross-sectional study from December 2020 to December 2021 on 6125 clinical samples referred to the Pasteur Institute of Iran that were suspected to be infected with TB. American Thoracic Society and Infectious Diseases Society of America (ATS/IDSA) guidelines were used to identify NTM isolates (Aksamit et al. 2007). As clinically relevant to define NTM pulmonary disease, we included patients with at least two positive cultures from sputum samples and/or bronchoalveolar lavages (BALs). Only those that had three adequate sputum samples collected in consecutive mornings were included in this study. The other samples were including skin, urine, pus, joints, lymph nodes, and soft tissues. Löwenstein-Jensen (LJ) medium was used to culture all samples after decontamination with N-acetyl-L-cysteine and sodium hydroxide (Kent 1985).

### Identification of NTM strains using phenotypic and genotypic tests

Based on Centers for Disease Control (CDC) procedures, phenotypic tests for isolation of NTM strains included macroscopic and microscopic morphological characteristics, growth rate on LJ medium, growth at 25 °C, 32 °C, 37 °C, and 42 °C, Tween-80 hydrolysis, arylsulfatase, urease, tellurite reduction, nitrate reduction, semiquantitative catalase production, and salt tolerance (Kent 1985). Following the manufacturer's instructions, DNA was isolated from bacteria using a Proba-NK DNA extraction kit (DNA-Technology Company, Moscow, Russia). In positive cultures, insertion sequence 6110 (IS6110)-PCR (123 bp) was applied to differentiate MTC and NTM species (McKibben et al. 2016). As a primary method of detection, heat shock protein 65 (*hsp65*) fragments of 441 bp, followed by *16S rDNA* and *rpoB*, were used as multilocus sequence typing (MLST) (Telenti et al. 1993). The ABI Automated Sequencer (Applied Biosystems, Foster City, CA, USA) was used to sequence two highly conserved genes, *16S rDNA* (nearly 1500 bp) and *rpoB* (750 bp) genes from NTM isolates detected up to Mycobacterial species level (Adékambi et al. 2003; Kämpfer et al. 1991).

### Drug susceptibility testing (DST)

According to the guidelines from the Clinical and Laboratory Standards Institute (CLSI), the broth microdilution method was used to determine the minimum inhibitory concentrations (MICs) of each drug. A wide range of drugs were tested including isoniazid, rifampicin, ethambutol, streptomycin, ethionamide, amikacin, ofloxacin, ciprofloxacin, and capreomycin. The drug was diluted in Mueller–Hinton broth with 5% oleic acid, albumin, dextrose and catalase and serially double diluted between 0.06 and 512 mg/L. Each culture was incubated aerobically at 37 °C after inoculation. The growth of rapidly growing mycobacteria (RGM) was evaluated on days 3 and 7 and that of slowly growing mycobacteria (SGM) was evaluated weekly for up to four weeks. MICs are defined as the lowest concentration of an antimicrobial agent that inhibits visible growth. The CLSI guidelines were used to assign susceptible, moderately susceptible, and resistant breakpoints (Woods et al. 2011).

### Statistical analysis

The analyses of all clinical data and demographic characteristics were carried out using SPSS version 24.0 (2016; IBM Corp., Armonk, NY, USA). Statistics were considered significant at a P-value of 0.05 with two tailed tests. The Shapiro–Wilk test was initially used to verify the normality of continuous variables. For determining significant associations between qualitative and continuous variables, Fisher’s exact test/χ2 and Mann–Whitney U-test were used, respectively. Means were reported for variables with continuous distributions.

## Results

### Typical characteristics of the patients

**Fig. 1:**
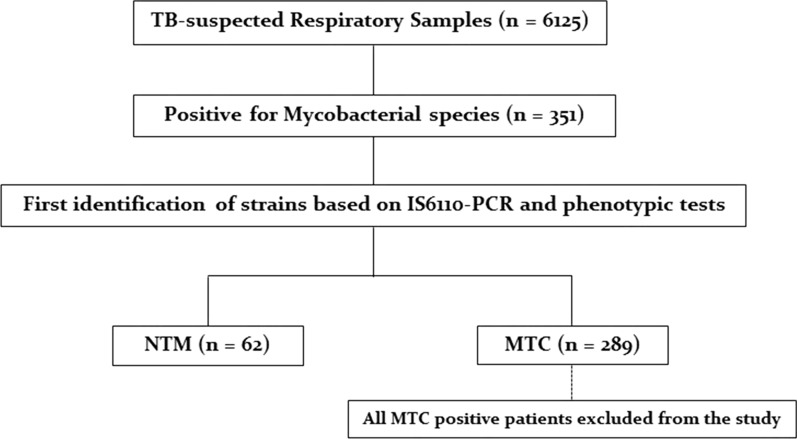
Population study flowchart. TB, *tuberculosis*; MTC, *Mycobacterium tuberculosis* complex; *NTM* nontuberculous mycobacteria

**Fig. 2:**
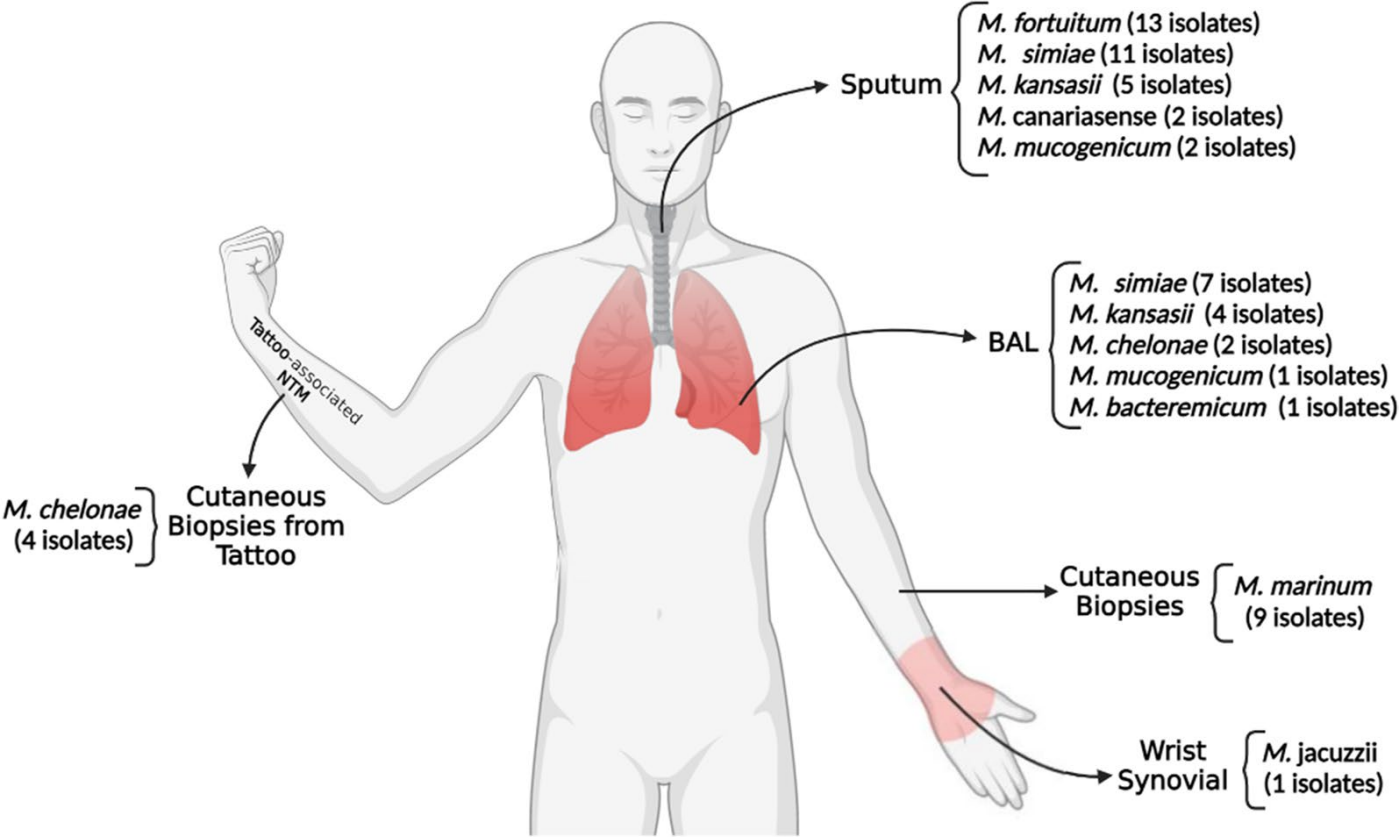
Overview of sample types used in collecting NTM isolates

An analysis of 6125 clinically samples of TB-suspected patients was conducted in this study by the culture method and 351 (5.7%) yielded acid-fast bacteria. By evaluating phenotypic and molecular data, 289 (82.3%) and 62 (17.7%) subjects were identified as *M. tuberculosis* complex (MTC) and NTM strains, respectively (Fig. 1). A summary of the baseline demographic characteristics of NTM patients is shown in Table 1.  In brief, 36 (61.3%) of the samples were sputum, and 15 (17.7%) were BALs collected from pulmonary sites. Further, 13 (21.0%) samples of suspected lesions were biopsied. An overview of the types of samples used to collect different isolates of NTM is presented in Fig. 2. Symptoms included coughing, sputum, fever, weight loss, and night sweating. Among the NTM patients, the average age was 53.2 ± 11.6 years old and the number of men and women was 35 (56.5%), 27 (43.5%). The smear was positive in 38 (21.3%) samples. A total of 14 (22.6%), 10 (16.1%), 8 (12.9%), 6 (9.8%), 5 (8.1%), and 4 (6.5%) had obesity, gastrointestinal diseases, human immunodeficiency virus (HIV), cystic fibrosis (CF), diabetes mellitus, and asthma, respectively.

**Table 1:** Clinical characteristic of patients infected with NTM isolates

Factors	NTM positive patients (n = 62)	*P*-value
Mean age ± SD	53.2 ± 11.6	0.072
*Gender*		0.125
Male	35 (56.5%)	
Female	27 (43.5%)	
*History of smoking*		0.752
Smokers	29 (46.8%)	
Non-smokers	32 (53.2%)	
*Symptoms*		0.042*
Cough	49 (92.5%)	
Sputum	45 (84.9%)	
Fever	45 (84.9%)	
Weight loss	35 (66.1%)	
Night perspiration	32 (60.4%)	
Gastroesophageal	26 (49.1%)	
Dyspnea	18 (33.9%)	
Hemoptysis	12 (22.6%)	
*Underlying disease*		0.052
Obesity	14 (22.6%)	
Gastrointestinal diseases	10 (16.1%)	
HIV	8 (12.9%)	
Cystic Fibrosis	6 (9.8%)	
Diabetes mellitus	5 (8.1%)	
Asthma	4 (6.5%)	
*AFB smear microscopy*		0.457
Positive	38 (21.3%)	
Negative	24 (38.7%)	
*Radiographic findings*		0.038*
Bronchiectasis	30 (48.4%)	
Infiltrate	22 (35.5%)	
Cavitary	15 (24.2%)	
Consolidation	13 (21.0%)	
*NTM isolates*		
*M. simiae*	18 (29.0%)	
*M. fortuitum*	13 (21.1%)	
*M. kansasii*	9 (14.5%)	
*M. marinum*	9 (14.5%)	
*M. chelonae*	6 (9.7%)	
*M. mucogenicum*	3 (4.8%)	
*M. canariasense*	2 (3.2%)	
*M. bacteremicum*	1 (1.6%)	
*M. jacuzzii*	1 (1.6%)	

*Statistically significant (< 0.05)

*HIV* human immunodeficiency virus, *NTM* nontuberculous mycobacteria, *AFB* acid fast bacilli, *SD* standard deviation

### Identification of NTM isolates by phenotypic tests

Twenty-seven (43.5%) and 35 (56.5%) of the 62 isolates were RGMs and SGMs, respectively. A phenotypic analysis revealed that *M. simiae* (17 isolates) and *M. fortuitum* (12 isolates) were the most prevalent strains, followed by *M. kansasii* (9 isolates), *M. marinum* (9 isolates), and, *M. chelonae* (5 isolates). The phenotypic tests identified 52 (83.9%) strains of 62 isolates, while the rest could not be identified.

### Identification of NTM isolates by molecular tests

A MLST molecular test (*hsp65*, *rpoB*, and *16S rDNA*) determined that *M. simiae* (18 isolates), *M. fortuitum* (13 isolates), *M. kansasii* (9 isolates), *M. marinum* (9 isolates), *M. chelonae* (6 isolates), *M. mucogenicum* (3 isolates), *M. canariasense* (2 isolates), *M. bacteremicum* (1 isolates), and *M. jacuzzii* (1 isolates) were among NTM isolates (Table 1).

### The clinical significance of pulmonary NTM isolates

Table 2 reports patients’ characteristics of pulmonary NTM isolates. NTM isolates were not significantly associated with mean age, smoking history, and underlying disease. However, there was a correlation between NTM isolates and gender and radiographic findings. The *M. simiae* group had 9 (50.0%), 4 (22.2%), 3 (16.6%), 1 (5.6%), and 1 (5.6%) patients with obesity, HIV, CF, diabetes mellitus, and asthma, respectively. In this group, there was a high frequency of infiltrates. The mean age of patients with *M. simiae* infection was older and they were mostly female (69.2%). Isoniazid, rifampicin, ethambutol, and streptomycin were resistant to the majority of *M. simiae* strains, while amikacin, ofloxacin, and ciprofloxacin were susceptible.

**Table 2:** Patients properties based on pulmonary NTM isolates

Factors	*M. simiae* (n = 18)	*M. fortuitum* (n = 13)	*M. kansasii* (n = 9)	*M. mucogenicum *(n = 3)	*M. chelonae* (n = 2)	*P*-value
Mean age ± SD	51.4 ± 11.8	61.2 ± 12.5	45.2 ± 10.1	47.1 ± 10.8	48.2 ± 11.1	0.132
*Gender*						0.012*
Male	7 (38.9%)	4 (30.8%)	5 (55.6%)	2 (66.7%)	1 (50.0%)	
Female	11 (61.1%)	9 (69.2%)	4 (44.4%)	1 (33.3%)	1 (50.0%)	
*History of smoking*						0.678
Smokers	8 (44.4%)	9 (69.2%)	6 (66.7%)	2 (66.7%)	2 (100.0%)	
Non-smokers	10 (55.6%)	6 (30.8%)	3 (33.3%)	1 (33.3%)	0 (0.0%)	
*Underlying disease*						0.359
Obesity	9 (50.0%)	1 (7.7%)	2 (22.2%)	0 (0.0%)	0 (0.0%)	
Gastrointestinal diseases	0 (0.0%)	9 (69.2%)	1 (11.1%)	0 (0.0%)	0 (0.0%)	
HIV	4 (22.2%)	1 (7.7%)	1 (11.1%)	0 (0.0%)	0 (0.0%)	
Cystic Fibrosis	3 (16.6%)	0 (0.0%)	1 (11.1%)	1 (33.3%)	1 (50.0%)	
Diabetes mellitus	1 (5.6%)	1 (7.7%)	2 (22.2%)	1 (33.3%)	0 (0.0%)	
Asthma	1 (5.6%)	1 (7.7%)	2 (22.2%)	1 (33.3%)	1 (50.0%)	
*Radiographic findings*						0.029*
Bronchiectasis	6 (33.3%)	11 (84.6%)	2 (22.2%)	2 (66.7%)	1 (50.0%)	
Infiltrate	10 (55.6%)	5 (38.5%)	3 (33.3%)	1 (33.3%)	1 (50.0%)	
Cavitary	2 (11.1%)	3 (23.1%)	8 (88.9%)	0 (0.0%)	1 (50.0%)	
Consolidation	6 (33.3%)	4 (30.8%)	2 (22.2%)	1 (33.3%)	0 (0.0%)	

*Statistically significant (< 0.05)

*HIV* human immunodeficiency virus, *NTM* nontuberculous mycobacteria, *SD* standard deviation

Among the *M. fortuitum* positive group, chest radiography was also most frequently interpreted as having bronchiectasis (84.6%). Among the patients, there were 1 (7.7%), 9 (69.2%), 1 (7.7%), 1 (7.7%), and 1 (7.7%) patients with obesity, gastrointestinal diseases, HIV, diabetes mellitus, and asthma, respectively. It was found that 69.2% of the patients who were infected suffered from gastroesophageal disease, including chronic vomiting and achalasia. There were several major symptoms observed in these patients, including cough, sputum, fever, and weight loss. There was a high level of resistance to isoniazid, rifampicin, ethambutol, and streptomycin among the *M. fortuitum* strains, while amikacin was susceptible to most of them.

There were 2 (22.2%), 1 (11.1%), 1 (11.1%), 1 (11.1%), 2 (22.2%), and 2 (22.2%) patients with obesity, gastrointestinal diseases, HIV, CF, diabetes mellitus, and asthma, respectively, among the *M. kansasii* group. Chest radiography results showed cavitary lesions in all patients. In this group of patients, hemoptysis was more common. It was found that most of the strains of *M. kansasii* were susceptible to isoniazid, rifampicin, ethambutol, and streptomycin, and resistant to amikacin, ofloxacin, and ciprofloxacin.

## Discussion

A key component of controlling and preventing tuberculosis is the isolation and detection of mycobacteria. We enrolled 6125 presumptive pulmonary tuberculosis patients in our study over one year, and 62 of them (17.7%) tested positive for NTM. In a similar study conducted in Iran during 2019, 53 (11.1%) out of 478 suspected pulmonary tuberculosis patients were found to be infected with NTM, which is comparable to our findings (Mortazavi et al. 2019). This study showed that the most common NTM isolates were *M. fortuitum*, followed by *M. simiae*, *M. kansasii*, *M. gordonae*, and *M. conceptense*. Aside from that, a variety of studies have been conducted on the prevalence of NTM infections, in particular *M. avium-intracellulare* (MAC) infection, *M. abscessus*, *M. chelonae*, *M. fortuitum*, and *M. kansasii* (Chiang et al. 2021; Zhou et al. 2021). Our study revealed that in the majority of cases, *M. simiae*, *M. fortuitum*, *M. kansasii* and *M. marinum* were detected. Our isolates also include *M. chelonae*, *M. canariasense*, and *M. jacuzzii,* all of which are rarely reported.

A real infection or colonization could be indicated by presence of *M. simiae* in respiratory samples (Nasiri et al. 2018). *M. simiae* infections were most common in females and older patients than other NTM isolates in the current study. In general, men are more likely than women to suffer pulmonary disease from NTM species except for *M. abscessus*, *M. chelonae*, and *M. simiae*, a finding in line with ours (Coolen-Allou et al. 2018; Jabbour et al. 2020). We found that the *M. simiae* positive cases in our study had obesity, HIV, cystic fibrosis, diabetes mellitus, and asthma as underlying diseases. There was a 21% incidence of clinical disease among *M. simiae* isolates, according to early surveillance reports, but other reports have shown a much lower rate (Griffith et al. 2007). There may be clinical and radiologic similarities between *M. simiae* and tuberculosis (Dezhkhi et al. 2021). As part of the present study, *M. simiae* isolated from TB patients receiving anti-TB drugs were originally diagnosed as multidrug-resistant. As a consequence, DST should be performed on every *M. simiae* isolate (Dezhkhi et al. 2021). The present study noted that *M. simiae* isolates had a wide range of susceptibility to amikacin, ofloxacin, and ciprofloxacin, unlike Van Ingen et al. who reported susceptibility to amikacin (14–40%) and ciprofloxacin (33–62%) (van Ingen et al. 2012). Several geographical regions show different susceptibility profiles for *M. simiae*, emphasizing the need to test susceptibility before starting treatment in these regions. Despite this, little is known about in vitro susceptibility and treatment response (Hamieh et al. 2018).

In our study, *M. fortuitum* were also frequent isolated NTMs from respiratory samples. *M. fortuitum* is the most frequently isolated RGM in Iran’s environmental and clinical samples (Arfaatabar et al. 2021; Ayoubi et al. 2021). In accordance with other studies, most patients had an underlying disease such as gastrointestinal diseases, HIV, cystic fibrosis, or diabetes mellitus. Additionally, the radiographic findings of these patients showed a tendency toward bronchiectasis. Structured lung disease appears to be the most common cause of *M. fortuitum* pulmonary infection (Irandoost et al. 2018). The ATS/IDSA guideline states that *M. fortuitum* is occurring in 15% of patients with pulmonary disease due to RGM, which is often observed as a pathogen in conditions such as chronic vomiting, achalasia, and exogenous lipoid pneumonia. As a result of most research on NTM pulmonary disease as well as esophageal disorders, it is discovered that most of the patients had achalasia and lung infection by *M. fortuitum* (Hadjiliadls et al. 1999; Jamal and Hammer 2022). As seen in the current study, most *M. fortuitum* infected patients had gastroesophageal disease and achalasia, which contributes significantly to *M. fortuitum* pulmonary disease. A minimum of two positive cultures were obtained by each *M. fortuitum* positive patient (Abraham 2007). *M. fortuitum* lung disease is rare, but it could potentially be the cause of disease in two of three respiratory sample cultures, according to several studies observing *M. fortuitum* isolation. As a result of our study, *M. fortuitum* was found to be one of highly pathogenic in respiratory samples. As shown by DST results against *M. fortuitum* isolates, this isolate was susceptible to amikacin as well as intermediate to ofloxacin, ciprofloxacin, and capreomycin. Drugs have generally been more effective against the *M. fortuitum* group than other RGM species. There is usually a high susceptibility or intermediate susceptibility to doxycycline, fluoroquinolones, sulfonamides, and macrolides in these bacteria (Brown-Elliott and Philley 2017). Nevertheless, it is suggested that treatment with antibiotics for most *M. fortuitum* positive patients may not be necessary (Mortazavi et al. 2019). It may be appropriate to use less invasive treatment strategies for this group of patients because *M. fortuitum* infection can be seen as colonization or a transient infection.

This study found *M. kansasii* to be the third most frequently occurring pulmonary NTM. As a result of its clinical and antigenic properties, it is similar to *M. tuberculosis*, whose prevalence is high in polluted cities (Nour-Neamatollahie et al. 2017). There were a variety of radiographic findings for *M. kansasii* pulmonary disease in this study. The most common radiographic feature was a cavitary lesion. The prevalence of *M. kansasii* pulmonary disease cavitation has been reported to be 75–96% in several studies (Bakuła et al. 2018). In radiographic terms, *M. kansasii* pulmonary disease has characteristics that are similar to tuberculosis. It is recommended to treat *M. kansasii* pulmonary disease with a combination of isoniazid, rifampin, and ethambutol for at least 12 months after a negative sputum result. Our study also found the same results with DST for *M. kansasii* pulmonary disease as other studies. Ciprofloxacin showed the highest resistance to *M. kansasii* isolates, followed by isoniazid and rifampin (Davari et al. 2019). As in our study, Shitrit et al*.* also showed that *M. kansasii* isolates had the strongest resistance to ciprofloxacin and the strongest sensitivity to rifampin and ethambutol (Shitrit et al. 2007).

A higher rate of hemoptysis was observed in our group patients with *M. kansasii* than in other studies. Hemoptysis in lung infections may be associated with endobronchial disease and may cause bronchial vessel disintegration by cavitation (Bakuła et al. 2018; Griffith et al. 2007). The cavitation rate associated with the *M. kansasii* pulmonary infection is reported at 57%, which is similar to our study in terms of the severity of the disease. However, there is no practical information regarding the relative incidence of endobronchial disease. In patients with other types of NTM infections, cavitations were far less common (Davari et al. 2019; Liu et al. 2019; Shitrit et al. 2008). A higher number of patients had hemoptysis, dyspnea, and cough, whereas fewer had fever or night sweats. The results in this study were inconsistent with those found in the Shitrit et al*.* report (Shitrit et al. 2007). The short period between the onset of symptoms and diagnosis may have caused these discrepancies.

One more isolated NTM from TB-suspected patients is *M. marinum*. As a closely related species to *M. tuberculosis*, *M. marinum* causes extrapulmonary mycobacterial infections ranging from cutaneous lesions to disseminated infections in immunocompromised individuals (Canetti et al. 2022; Tarashi et al. 2022). In our study, this bacterial agent was found in cutaneous biopsies of patients. It is most common for cutaneous bacterial infections to be caused by ubiquitous potential pathogens, while NTMs are rarely involved. It is reported, however, that NTM infections are becoming more prevalent over time (Sander et al. 2018). Cutaneous infections caused by NTMs were most prevalent with MAC (68%) and *M. marinum* (24%) (Lee et al. 2010). In Canada, *M. marinum* was found to occur in 0.08 cases per 100,000 people (Sander et al. 2018). As compared with France where there are 0.04 cases per 100,000 and the United States where there are 0.05 to 0.27 cases per 100,000, this is a lower incidence (Yu et al. 2016). *M. ulcerans* and *M. marinum* nearly always cause cutaneous infection through direct inoculation, unlike most NTMs that cause disseminated diseases (Kothavade et al. 2013; Lee et al. 2010). Among the factors that increase the risk of *M. marinum* infection is exposure to freshwater and saltwater, swimming in pools that are unchlorinated and owning and handling fish tanks (Hashish et al. 2018; Sia et al. 2016). According to our results, occupational exposure and fish tank cleaning are the most common sources of exposure. Adults with upper extremity involvement are typically affected (Sander et al. 2018). Among the antibiotics tested, ciprofloxacin showed the highest resistance to *M. marinum* isolates and the susceptibility to rifampin and ethambutol.

It is rare to find *M. chelonae*, *M. canariasense*, and *M. jacuzzii* in various samples of TB-suspected patients. The *M. chelonae* species are ubiquitous in the environment and have been found in soil, water, and aquatic animals (Akram et al. 2017). Infections of the extremities, such as cellulitis and abscesses, are commonly associated with this bacterial agent. *M. chelonae* also causes catheter-related infections and post-surgical infections after implant placement, transplantation, and sclerotherapy injections (Jones et al. 2019; Nakamura et al. 2019). Our results show that 50% of *M. chelonae* is isolated from cutaneous biopsy samples of tattoo. Despite the limited literature on mycobacterial infections complicating tattoos, tattoo-associated infections occur frequently (Drage et al. 2010). In an evaluation in 2003, an unclassified mycobacterial species was identified in an erythematous nodule associated with a tattoo (Wolf and Wolf 2003). De Quatrebarbes et al*.* described an outbreak of *M. chelonae* associated with tattoos that resulted in multiple, pruritic papules and pustules (De Quatrebarbes et al. 2005). A number of antibiotics have demonstrated in vitro susceptibility to *M. chelonae*, including clarithromycin, amikacin, tobramycin, linezolid, and tigecycline (Borek et al. 2022). For localized infections caused by *M. chelonae* while waiting for susceptibility results, clarithromycin and azithromycin are useful oral agents. Nevertheless, it has been well documented that patients with *M. chelonae* are resistant to clarithromycin (Jhaveri et al. 2020; Wallace et al. 1993). Amikacin demonstrated the resistance to *M. chelonae* isolates, whereas rifampin, isoniazid, and ethambutol showed the lowest susceptibility.

It is also interesting to note that *M. canariasense* has also been isolated from respiratory samples in the present study. Patients with immunocompromised immune systems were reported to develop skin infections and subcutaneous fat infections from *M. canariasense* isolates (Michienzi et al. 2019). There have also been few studies that confirm that this isolate is a pulmonary pathogen (Kim et al. 2012; Lee et al. 2014). The presence of *M. conceptionense* was only detected in two patients with pulmonary disease in one study in Iran (Shojaei et al. 2011). The second case of *M. conceptionense* in Iran was found in a 37 year-old male with HIV infection and 51 year-old male with diabetes mellitus (Mortazavi et al. 2019). It was interesting to note that both patients had underlying diseases, and that DST showed that they were susceptible to ofloxacin, ciprofloxacin, and amikacin, as well as intermediate to isoniazid, rifampicin, and ethionamide, which is in line with our findings. The results concur with Kim et al. s study, which suggested *M. conceptionense* can cause pulmonary disease, despite being a rare NTM (Kim et al. 2012).

Additionally, we detected *M. jacuzzii* in biopsy samples which is a very rare NTM. NTM tenosynovitis most commonly affects the hand and wrist due to the redundancy of tissues and synovial fluid. In most cases, these conditions are caused by mycobacteria, especially *M. marinum*, which are slowly growing. In Israel, breast implants first showed evidence of *M. jacuzzii* isolation in 2003 (Rahav et al. 2006). Using phenotypic and genotypic tests on wrist synovial samples, we described our experience in detecting *M. jacuzzii* in Iran (Sakhaee et al. 2020). The antimycobacterial agents that are active against *M. jacuzzii* are limited. There were three antibiotics that were susceptible to this bacterium including amikacin, levofloxacin, and ethambutol. Generally, our study was limited by inadequate follow-up information. In addition, chest radiography was the main focus of the radiologic evaluation, not a CT scan.

In conclusion, the presence of nine NTM species was detected in samples taken from patients suspected of having TB. Most commonly found NTM species were *M. simiae* and *M. fortuitum*, followed by *M. kansasii* and *M. marinum* with a variety of radiographic findings, clinical symptoms, and drug resistance. Additionally, we isolated *M. chelonae*, *M. canariasense*, and *M. jacuzzii*, all of which have seldom been reported before. Further research is needed on the epidemiology of NTM infections in Iran with the aim of increasing Iranian physicians’ knowledge regarding the diagnosis and treatment of NTM isolates.

## Data Availability

The data used to support the findings of this study are included within the article and supplementary file. The nucleotide sequence data are available in the GenBank databases under the accession numbers OP580654 to OP580841 for the *16S rDNA*, OP793506 to OP793567 for *hsp68*, and OP793568 to OP793629 for *rpoB* genes.
